# Effect of Acute Judo Training on Countermovement Jump Performance and Perceived Fatigue among Collegiate Athletes

**DOI:** 10.3390/ijerph192417008

**Published:** 2022-12-18

**Authors:** Chien-Chun Chang, Tzu-Yi Chen, Chia-Luan Wu, Pi-Yen Ho, Chieh-Ying Chiang

**Affiliations:** 1 Graduate Institute of Athletics and Coaching Science, National Taiwan Sport University, Taoyuan 333, Taiwan; 2 Department of Athletic Training and Health, National Taiwan Sport University, Taoyuan 333, Taiwan; 3 Department of Sports Training Science-Combats, National Taiwan Sport University, Taoyuan 333, Taiwan

**Keywords:** neuromuscular fatigue, modified rating of perceived exertion, sport performance

## Abstract

This study focused on the effect of acute Judo training on countermovement jump (CMJ) performance and perceived fatigue among a group of highly trained collegiate judo athletes. Twenty male judo athletes participated in this study (age: 20.65 ± 1.22 years, weight: 84.17 ± 28.45 kg). Participants were assessed for CMJperformance changes before, immediately after (0 h), 12 h after, and 24 h after judo training (JT) using unloaded CMJ(CMJ_unloaded_) and loaded CMJ(CMJ_loaded_). All the jumps were performed on a force plate, and the force–time curves were collected for further analysis. Respondents’ perceptions were evaluated using the modified rating of perceived exertion (mRPE) before, after (0 h), 12 h, and 24 h after JT. CMJparameters were analyzed at four measured points using a one-way repeated analysis of variance. Effect sizes (ES) and percentage changes before versus 24 h after JT were calculated for comparison. Associations between the CMJparameters and mRPE were analyzed using the Pearson product–moment correlation. The ratio of flight time to contact time significantly decreased, whereas the eccentric duration, concentric duration, and total duration significantly increased (*p* < 0.05) in both CMJs 24 h after JT. Compared with CMJ_unloaded_, CMJ_loaded_ had a significantly lower (*p* < 0.05) flight time, jump height, peak velocity, and peak power. The mRPE and CMJ_loaded_ peak velocity showed moderate- to high-level negative correlation results both 0 and 24 h after training (*r* = −0.543, *p* < 0.05; *r* = −0.479, *p* < 0.05). In this study, we only observed the effect of fatigue on the neuromuscular (NM) system 24 h after JT. CMJ_loaded_ height may help to better determine fatigue state compared with CMJ_unloaded_. According to the results, the neuromuscular effects of fatigue were not observed until 24 h after a single high-intensity training. Therefore, when arranging high-intensity special training or strength and conditioning training, one should reduce the volume of training appropriately to avoid fatigue accumulation and reduce the risk of sports injuries.

## 1. Introduction

Acute mechanical fatigue can be caused by training and can affect athletes performance. Continual high-intensity competition and the lack of a sufficient recovery period can lead to the accumulation of fatigue [1]. Monitoring athletes training can allow for coaches and sports scientists to better understand athletes physical and psychological state and adjust their training programs accordingly [2]. Moreover, it can allow for athletes to properly recover or improve their performance [3,4]. Recent research has showed that athletic jump ability, objective data, and subjective response to perception and cognition could provide information on athlete fatigue [3].

Judo is a high-intensity interval sport and requires athletes to possess considerable technical and physical skills [5,6]. Each judo competition has a time limit of 5 min [6]. It takes an average of 20–30 s for each offensive and defensive action, with an approximately 5–10 s interval of pauses [6]. This continuous, high-intensity competition and short recovery time lead to the accumulation of fatigue [7].

Furthermore, elite and sub-elite judo athletes exhibit considerable differences in strength levels [8]. For example, elite judo athletes perform a one-repetition maximum back-squat significantly better than sub-elite judo athletes. In terms of jump performance, elite judo athletes exhibited high power output, indicating that elite judo athletes showed a higher muscle strength and power output of the lower limbs [9,10]. Some lower limb actions involve the stretch-shortening cycle (SSC) and are performed by leveraging the opponent’s body weight, as in several judo throwing techniques [9,11,12]. Therefore, the lower limbs’ ability to execute fast and forceful actions when bearing weight may be related to judo-specific performance.

However, most sports scientists use questionnaires and neuromuscular function tests to assess fatigue [1,3,13]. More recently, research has shown that the vertical jump test was easier and faster, enhanced athletes’ confidence, led to less fatigue, and provided real-time feedback [14]. Additionally, questionnaires were used to assess players subjective responses to training or competition [15,16]. A modified rating of perceived exertion (mRPE) can be used to monitor training by examining simple markers of training volume and intensity [17,18,19]. Previously, most studies observed that neuromuscular system function can be used to monitor fatigue [20,21]. As the neuromuscular system accumulates fatigue, changes in the muscle SSC are involved in most sporting movements at different velocities and intensities [4,14,22,23].

One approach to assessing an individual’s dynamic strength is conducting load and unload countermovement jump (CMJ_loaded_ & CMJ_unloaded_) tests [24,25]. The CMJ test has also been used to evaluate judo performance. Previous studies have shown that CMJ jump height (JH) is correlated with the likelihood of winning a competition (*r* = 0.69). The better the athlete muscle performance and CMJ_loaded_ JH (compared with CMJ_unloaded_ height), the more negligible the difference in force produced during the jump movement [24,25]. Athletes with better muscle strength exhibit a relatively high-power output in the lower limbs under body resistance. In some instances, athletes must perform high-power actions with the external load state.

To the best of our knowledge, no research has been conducted on the characteristics of judo techniques that require load bearing or has discussed the CMJ_loaded_ and CMJ_unloaded_ with the features of lower limb muscle strength, even as a method to monitor fatigue. Therefore, the purpose of this study was to explore the results of CMJ and mRPE performance by outstanding judo players in junior colleges after high-intensity special training to understand the relationship between various subjective and objective measurement variables, and to try to find potential indicators that can be used in fatigue-monitoring.

## 2. Materials and Methods

At the training center of the University’s judo team, the test inspector verbally explained the recruitment information to the male athletes. Participants gave their written informed consent. Participants were required to understand and be familiar with the CMJtest to avoid affecting the results. The testing process was divided over two days split into three sessions: A, B, and C. Day 1: session A was conducted at 6:30 a.m. with a CMJ_unloaded_ test, CMJ_loaded_ test, and a series of mRPE; and session B was conducted after high-intensity training with two types of CMJ test. Day 2: session C was conducted 12 and 24 h after training with *CMJ_unloaded_* and *CMJ_loaded_* tests [26,27].

### 2.1. Participants

Twenty (*n* = 20) male judo athletes (age: 20.65 ± 1.22 years, weight: 84.17 ± 28.45 kg) voluntarily participated in this study. Participants were required to meet the inclusion criteria of having at least 6 years of judo training experience, 6 months of strength training experience, and being familiar with the experimental procedures and risks. Participants could not have injuries in the lower limbs at the time of testing or have a history of major lower limb injuries, such as anterior cruciate ligament injuries or broken bones. This study was approved by the Research Ethics Committee at the National Taiwan University. All the participants provided informed consent before participation, and all the testing procedures were explained to the participants.

### 2.2. Judo Training

The judo training (JT) program followed previous research [28,29] and discussions with judo coaches. The JT was designed according to anaerobic physiological system training under continuous maximum effort and simulates an actual judo competition, which may induce physiological responses such as a short-term high heart rate, oxygen uptake, and lactate concentration [6,28,29]. The training included 15 mins of warm-ups (dynamic warm-up and Uchi-Komi), followed by 2 mins of pressing exercises (Ne-Waza), 4 mins of squatting exercises, and 1 min of falling movements (Naga-Komi), with 2 min rests between sets for a total of eight cycles; and four sets of rope climbing. The entire training lasted approximately 120 mins. Post-training mRPE can be collected as fast as 10 mins after training a session with no loss of measurement quality [30].

### 2.3. Countermovement Jump Assessment

Each test was led by the same test personnel, who performed a 5–10 min dynamic warm-up. The warm-up content included 25 repetitions of a jumping jack, 30 repetitions of high knee run, 15 repetitions of air squat, 20 repetitions of side lunges, and 5 repetitions of CMJ. Before the formal test, the subject performed one trial jump to familiarize them with the force plate. The CMJ_unloaded_ and CMJ_loaded_ test interval lasted 10–15 min. Participants performed a CMJ_unloaded_ and CMJ_loaded_ on a portable force plate (9260AA, Kistler Group, Winterthur, Switzerland) with sampling at 1000 Hz. The participants were instructed to stand with their feet shoulder-width apart and their hands positioned on a polyvinyl chloride (PVC) bar. A 20 kg barbell was placed across their shoulders, and they were instructed to jump as high as possible [31]. Ground reaction force data were collected using a USB data acquisition system (Type 5695B, Kistler Group, Winterthur, Switzerland). Data were analyzed using Bioware software (Version 5.0.3; Bioware Software Type 2812A, Kistler Group, Winterthur, Switzerland) with the force (recorded in newtons [N]) determined in two CMJs [32]. All data is reported as the mean ± standard deviation. The CMJ parameters were flight time (FT), contact time (CT), ratio of flight time to contact time (FT: CT), jump height (JH), peak force (PF), peak power (PP), peak velocity (PV), eccentric duration (EccDur), concentric duration (ConDur), and total duration (TotDur). The kinetic parameters were normalized (absolute strength and bodyweight: 0.67) to exclude the effect of weight differences [24].

### 2.4. Modified Rating of Perceived Exertion Assessment

The participants provided subjective feedback on training according to the modified rating of perceived exertion (mRPE) before, immediately after (0 h), 12 h after, and 24 h after JT. To measure training intensity, the subject was asked to rate the intensity of the end 30 mins after completion of the JT. The training intensity assessment included a 0–10 conscious intensity score [33]. This was undertaken by asking them ‘How was your workout?’ and having them rate it against an mRPE [19,33]. Previous research indicated that the delay in asking the player/athlete to rate the intensity of the session ensures that the rating reflects the global intensity of the session [33,34].

### 2.5. Statistical Analysis

The test–retest reliability of the CMJ test was calculated using the intraclass correlation coefficient (ICC). A one-way repeated analysis of variance was used to determine the changes in CMJ performance before versus after training at four timepoints. Each pre-test and 24 h effect size (ES) was simultaneously calculated, and the change in rate percentage was calculated before and after. The effect size is the independent variable’s degree of influence on the dependent variable. According to the standard proposed by Cohen [35], ES ≤ 0 to 0.19 is a low effect, ES = 0.2 to 0.8 is a moderate effect, and ES ≥ 0.8 is a high effect (Table 1). The associations between unloaded and loaded CMJ parameters include FT, CT, FT: CT, JH, PF, PP, PV, EccDur, ConDur, and TotDur with the mRPE; these were analyzed using the Pearson product-moment correlation coefficient. The strength of the relationship was determined according to the Hopkins criteria: slight (*r* < 0.001), low (*r* = 0.1–0.2), moderate (*r* = 0.3–0.4), high (*r* = 0.5–0.6), very high (*r* = 0.7–0.8), near-perfect (*r* = 0.9), and perfect (*r* = 1.0) [36]. Significance was indicated at *p* < 0.05.

## 3. Results

**Figure 1:**
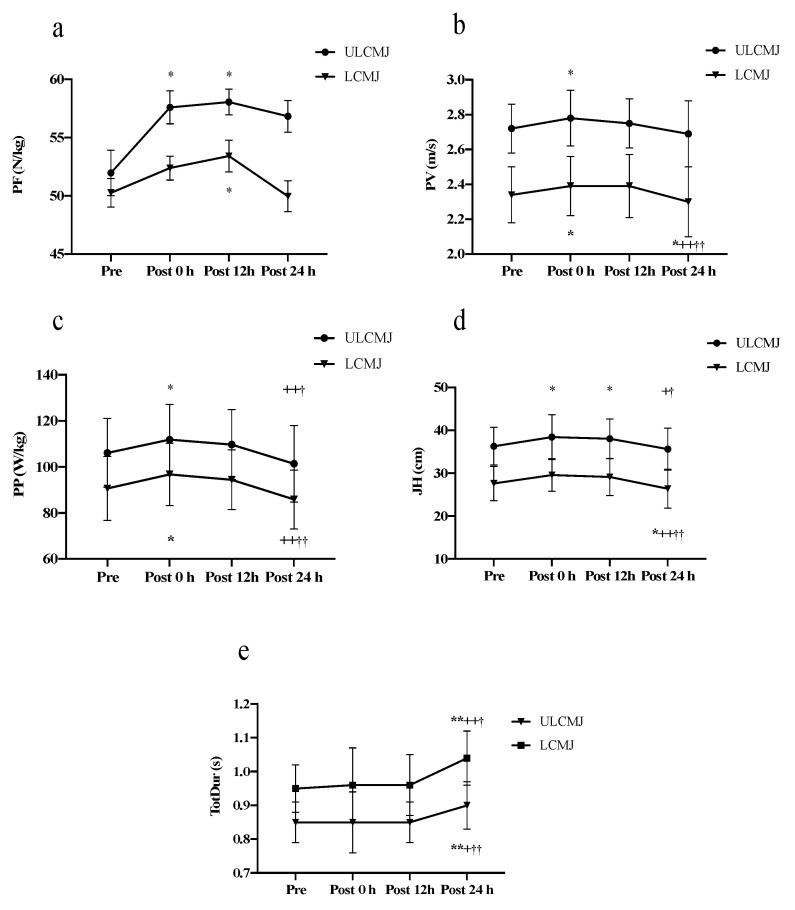
CMJ_unloaded_ and CMJ_loaded_ parameters at different points in time. *,** Significant compared with pre-test, *p* < 0.05, *p* < 0.001; +,++Significant compared with post-test at 0 h, *p* < 0.05, *p* < 0.001; †,†† Significant compared with post-test at 12 h, *p* < 0.05, *p* < 0.001. peak force, PF (**a**); peak power, PP (**b**); peak velocity, PV (**c**); jump height, JH (**d**); total duration, TotDur (**e**).

The results of the CMJ test of all the participants had high reliability, and most of the test variables had values between 0.90 and 0.99. A few variables, such as CT, PF, EccDur, and TotDur, ranged from 0.82 to 0.83. Previous studies have indicated that an ICC ≥ 0.75 indicates good reliability [37]. The mRPE score of 7.2 ± 1.67 indicated that the intensity of judo training was challenging for the participants; therefore, the judo training was high-intensity [38]. The CMJ_unloaded_ and CMJ_loaded_ related variables, specifically FT, CT, FT: CT, JH, PF, PP, PV, EccDur, ConDur, and TotDur, were compared at four time-points (before, immediately after (0 h), 12 h after, and 24 h after JT). Compared with before JT, out of the CMJ_unloaded_ variables, 24 h after training, the CT, EccDur, ConDur, and TotDur were significantly increased (*p* < 0.05), and only FT:CT was significantly decreased (*p* < 0.05). In the unloaded state, the jumping movement time of the variable considerably increased. Furthermore, in the CMJ_loaded_, the participants’ FT, FT:CT, JH, PP, and PV were significantly decreased (*p* < 0.05), and CT, EccDur, and ConDur were significantly increased (*p* < 0.05). Only PF showed no significant difference. Detailed results are shown in Table 1. Further comparisons of changes in PF, PP, PV, JH, and TotDur of CMJ_unloaded_ and CMJ_loaded_ were made at four timepoints (Figure 1). Significant increases were found for TotDur (CMJ_unloaded_, before: 0.854 ± 0.069 s, after 24 h: 0.909 ± 0.072 s, *p* < 0.001; CMJ_loaded_, before: 0.950 ± 0.076 s, after 24 h: 1.045 ± 0.087 s, *p* < 0.001). The CMJ_loaded_ JH (before: 0.276 ± 0.040 m, after 24 h: 0.264 ± 0.045 m, *p* < 0.05), PP (before: 90.62 ± 13.87 w/kg, after 24 h: 85.83 ± 12.79 w/kg, *p* < 0.05), and PV (before: 2.345 ± 0.17 m/s, after 24 h: 2.306 ± 0.20 m/s, *p* < 0.05) were significantly decreased (Table 1).

Table 2 showed the effect size and percentage increase and decrease of the vertical jump-related variables in the pre-test (Pre) and the post-test (24 h) periods at two time points. As shown in the table, the weight-bearing vertical jump was significantly higher in the effect size and percentage change of CT (ES = 1.40, 10.2%), FT:CT (ES = 1.22, 11.4%), EccDur (ES = 2.42, 11.9%), and TotDur (ES = 1.40, 10.4%), with more significant changes compared to unweighted vertical jumps.

However, the kinetics variables did not significantly improve in the CMJ_unloaded_ (JH, PF, and PP), whereas all but PF showed a significant decrease in the CMJ_loaded_. The significant difference in TotDur between CMJ_unloaded_ and CMJ_loaded_ was noteworthy. mRPE (whole, lower extremity) was correlated with CMJ_unloaded_ and CMJ_loaded_ at three timepoints (0, 12, and 24 h). By contrast, mRPE exhibited a significant negative correlation with PV at 0 and 24 h after training and a moderate to high level of negative correlation with CMJ_loaded_ (*r* = −0.543, *p* < 0.05; *r* = −0.479, *p* < 0.05, respectively). The perceived strength of the lower extremity (mRPE_leg_) exhibited a low-to-moderate level of negative correlation with JH, PP, and PV in the CMJ_unloaded_ and CMJ_loaded_ (*r* = −0.052 to −0.391) (Table 3).

## 4. Discussion

The study explored CMJ performance and perception changes caused by fatigue in elite judo athletes after high-intensity exercise. The main findings of the experiment were as follows: (1) neuromuscular fatigue had a significant effect on CMJ performance only 24 h after training; (2) JH, FT, PF, PP, and PV of CMJdid not necessarily change due to the influence of neuromuscular fatigue, but significantly prolonged the eccentric and concentric muscle contractions and the total movement time due to neuromuscular fatigue; (3) mRPE was negatively correlated with PV in the CMJ_unloaded_. Previous studies investigating neuromuscular fatigue on CMJ performance have demonstrated that CMJ performance declines immediately after training or competition, but most studies have focused on team sports [39,40,41,42,43]. However, the results demonstrated that CMJ performance only significantly decreased after 24 h, rather than immediately after training. The difference between the results of this study and previous studies may be due to the focus on different sports. Previous studies have involved participants who mainly played ball sports and required extensive room to move. Furthermore, judo athletes perform techniques on a 16 m × 16 m array of mats. Ball sports require extensive running, sprinting, or jumping, which require a higher SSC ability in the lower limbs. An increase or decrease in CMJ JH can be observed immediately after training or competition, whereas the proportion of combat types is lower. Therefore, the research results indicate different trends. In related studies, neuromuscular fatigue affected CMJ performance, and CMJ_unloaded_ JH significantly decreased or was maintained after training and competition [3,22,44,45]. JH is a commonly used fatigue indicator that is easy to interpret. According to this study and previous studies, the JH of the unloaded CMJ is not significantly reduced by fatigue. We surmised that we had to compensate for the neuromuscular effects of fatigue and that athletes would use different jumping strategies to achieve the same JH as in the pre-test. Additionally, the PF of CMJ_unloaded_ increased and the CMJ_loaded_ decreased, which did not indicate a significant difference, and presented inconsistent results compared to previous studies. The PF of the CMJ significantly decreased [22]; however, most studies showed no significant change in the PF of the CMJ after training or competition [14,39].

Although the PP of CMJ_unloaded_ was slightly but not significantly decreased, the PP of CMJ_loaded_ significantly decreased (*p* < 0.05). Previous studies have shown that neuromuscular fatigue leads to a significant decrease or no significant change in PP [1,14,22,39,46], which is consistent with our results. Jumping power is related to force and velocity; according to this study and previous studies, when fatigue occurs, the contraction time of lower limb muscles is prolonged to generate the same amount of force. Furthermore, the movement speed decreases, resulting in a decrease in power output. Neuromuscular fatigue significantly reduces speed. Although the PV decreased, albeit non-significantly, in the CMJ_unloaded_, the PV in the CMJ_loaded_ significantly decreased (*p* < 0.05), which is consistent with previous findings that fatigue significantly decreases PV [14,22,45]. A possible reason for the inferred speed reduction is the speed of muscle contraction. EccDur, ConDur, and TotDur significantly increased in both CMJ_unloaded_ and CMJ_loaded_ (*p* < 0.05). Consistent with previous findings, the increase in muscle contraction time may be related to neuromuscular contraction and jumping strategy [14,22,46]. According to the results of this study, contraction time may be one of the most effective monitoring indicators when observing neuromuscular fatigue. In addition, fatigue reduces sports performance and affects ones psychological state [4]. Questionnaires can be used to measure the onset of perceived fatigue, and mRPE can be used to assess fatigue status [1,3,45,47,48]. In this study, the PV of CMJ_loaded_ was significantly and negatively correlated with mRPE at 0 and 24 h (*r* = −0.543, *p* < 0.05; *r* = 0.497, *p* < 0.05, respectively) Therefore, mRPE is negatively related to movement speed when a load is borne. In technical judo moves, the athlete bears the opponents weight, and the correspondingly slower speed may affect their performance in competitions.

Overall, the results of this study and previous studies showed that JH, PF, PP, and PV may be affected by neuromuscular fatigue and may be decreased or not significantly changed. On the contrary, both previous studies and this study have shown that when neuromuscular fatigue occurs, this results in a marked increase in contraction time. Fatigue does not change strength performance but leads to noticeable changes in strategy. We speculate that a possible reason for this is that, when neuromuscular fatigue occurs, to achieve the same JH, different jumping strategies are used to increase movement and muscle contraction time. It should be noted that, although most of the variables obtained under both conditions (CMJ_unloaded_ and CMJ_loaded_) were negatively affected 24 h after a HIT judo training session, post 0 and 12 h a post-activation potentiation (PAP) effect was observed, which indicated a positive effect (increasing performance) in some variables, especially at 0, whilst others were still higher than pre-values at 12 h (moment). Therefore, it is recommended to observe the changes in athletes’ jumping strategies when monitoring fatigue during actual training or competition to effectively observe the generation of fatigue.

In addition to reducing exercise performance, fatigue is also related to psychological aspects [4], and the generation of perceived fatigue can be measured through questionnaires [3,47,49]. Previous studies suggested that mRPE can more acutely judge fatigue state [1,3,45,50]. In this study, mRPE was used to evaluate training fatigue. The results of this study showed that the CMJ_loaded_ PV was significantly negatively correlated with mRPE at 0h and 24h (*r* = −0.543, *p* <.05; *r* = 0.497, *p* <0.05, respectively), representing that the higher the mRPE, the slower the movement speed. This result may be an essential finding for judo sports. Technical judo movements need to be carried out under the opponent’s weight, and the slower speed may affect the results of the competition.

However, CMJ_unloaded_ and CMJ_loaded_ have been used to assess the dynamic performance of lower-extremity muscle strength. The CMJ_loaded_ can better evaluate the strengths and weaknesses of lower-extremity muscle strength [24]. In this study, this was used as a measure of neuromuscular fatigue. The CMJ_unloaded_ and CMJ_loaded_ both showed a decline 24 h after training. However, the weight-bearing vertical jump changed considerably, and the ES value was also significantly higher. CMJ_loaded_ is a more effective and sensitive method of monitoring neuromuscular fatigue and is consistent with the characteristics of judo techniques that require a high-power output under resistance to external loads. In the future, for combat sports such as judo, CMJ can be used to monitor fatigue. If the environment and equipment permit, CMJ_loaded_ can more clearly observe the generation of neuromuscular fatigue.

### Limitations

As the results are shown in this study, using a force plate to measure judo athletes’ CMJ performance, plus the subjective measures, such as mRPE, maybe the alternative approach (non-invasive) to understanding neuromuscular fatigue after acute JT. However, caution should be made when implementing the mRPE and interpreting the testing results. Other confounding variables, such as athletes’ diet and sleep, could also have a profound effect on measuring mRPE, even the extra physical activities were restricted during the experimental period. Future studies are warranted to investigate the potential mechanisms of neuromuscular fatigue after intense JT.

## 5. Conclusions

Few studies have investigated the application of CMJ to monitor fatigue in martial arts. This study presents the following conclusions and suggestions: (1) The neuromuscular effects of fatigue were not observed until 24 h after a single, high-intensity training session. Thus, when arranging high-intensity special training or strength and conditioning training, one should reduce the volume of training appropriately to avoid the accumulation of fatigue and reduce the risk of sports injuries. (2) CMJ_loaded_ is an effective neuromuscular fatigue monitoring method and is consistent with the characteristics of judo techniques that require high-output movements against an external load. (3) After JT, perceived fatigue is negatively correlated with PV performance; the mRPE questionnaire can help to identify perceived fatigue in training. (4) Future research is required on long-term training monitoring to explore neuromuscular fatigue recovery, which could assist sports coaches and physical trainers in planning periodization training.

## Figures and Tables

**Table 1:** Changes in Countermovement Jump Kinematics and Kinetic Parameters at Different Timepoints after Judo Training.

Variable	Pre		Post 0 h		Post 12 h		Post 24 h	
	CMJ_Unloaded_	CMJ_Loaded_	CMJ_Unloaded_	CMJ_Loaded_	CMJ_Unloaded_	CMJ_Loaded_	CMJ_Unloaded_	CMJ_Loaded_
FT (s)	0.544 ± 0.034	0.473 ± 0.034	0.559 ± 0.037 *	0.490 ± 0.031 **	0.556 ± 0.032 *^+^	0.486 ± 0.035 *	0.538 ± 0.036 ^+,†^	0.463 ± 0.039 *^,++,††^
CT (s)	0.880 ± 0.072	0.978 ± 0.077	0.877 ± 0.093	0.996 ± 0.11	0.872 ± 0.068	0.995 ± 0.094	0.93 ± 0.077 *^,+,††^	10.074 ± 0.088 **^,+,††^
FT: CT	0.623 ± 0.07	0.488 ± 0.061	0.646 ± 0.089 *	0.500 ± 0.077	0.644 ± 0.08	0.495 ± 0.074	0.583 ± 0.075 *^,++,††^	0.436 ± 0.065 **^,++,††^
JH (m)	0.363 ± 0.044	0.276 ± 0.040	0.384 ± 0.052 *	0.295 ± 0.038 **	0.380 ± 0.046 *	0.291 ± 0.043 *	0.356 ± 0.049 ^+,†^	0.264 ± 0.045 *^,++,††^
PF (N/kg)	51.97 ± 1.95	50.26 ± 1.23	57.59 ± 1.41 *	52.38 ± 1.02	58.06 ± 1.11 *	53.42 ± 1.36 *	56.83 ± 1.37	49.96 ± 1.33 ^+,†^
PP (W/kg)	106.13 ± 14.96	90.62 ± 13.87	111.82 ± 15.32 *	96.77 ± 13.51 *	109.72 ± 15.2	94.44 ± 12.99	101.37 ± 16.67 ^++,††^	85.83 ± 12.79 *^,++,††^
PV (m/s)	2.728 ± 0.141	2.345 ± 0.17	2.78 ± 0.169 *	2.398 ± 0.17 *	2.752 ± 0.146	2.394 ± 0.18 *^+^	2.695 ± 0.197 ^+^	2.306 ± 0.20 *^,++,††^
EccDur (s)	0.581 ± 0.058	0.633 ± 0.064	0.576 ± 0.083	0.653 ± 0.092	0.575 ± 0.058	0.652 ± 0.08	0.612 ± 0.064 *^,+,†^	0.705 ± 0.075 *^,+,†^
ConDur (s)	0.274 ± 0.025	0.317 ± 0.033	0.279 ± 0.027	0.316 ± 0.04	0.275 ± 0.027	0.316 ± 0.036	0.297 ± 0.025 **^,++,††^	0.340 ± 0.038 *^,+,††^
TotDur (s)	0.854 ± 0.069	0.950 ± 0.076	0.855 ± 0.093	0.969 ± 0.115	0.850 ± 0.068	0.968 ± 0.093	0.909 ± 0.072 **^,++,††^	1.045 ± 0.087 **^,+,††^

*,** Significant compared with pre-test, *p* < 0.05, *p* < 0.001; ^+^,^++^ Significantly compared with post-test at 0 h, *p* < 0.05, *p* < 0.001; ^†^,^††^ Significant compared with post-test at 12 h, *p* < 0.05, *p* < 0.001; flight time, FT; contact time, CT; flight time–contact time ratio, FT:CT; jump height, JH; peak force, PF; peak power, PP; peak velocity, PV; eccentric duration, EccDur; concentric duration, ConDur; total duration, TotDur.

**Table 2:** Comparison of pre-test and post-24-h effect size and rate of percentage change in countermovement jump parameters.

Variable	Effect Size (ES)		Rate of Change (%)	
	CMJ_Unloaded_	CMJ_Loaded_	CMJ_Unloaded_	CMJ_Loaded_
FT (s)	0.17	0.42 *	0.9% ^↓^	2.6% ^↓^
CT (s)	0.69 *	1.40 **	5.7% ^↑^	10.2% ^↑^
FT: CT	0.54 *	1.22 **	6.2% ^↓^	11.4% ^↓^
JH (m)	0.13	0.43 *	1.7% ^↓^	5.1% ^↓^
PF (N/kg)	0.64	0.05	9.4% ^↑^	0.6% ^↓^
PP (W/kg)	0.30	0.39 *	4.5% ^↓^	5.7% ^↓^
PV (m/s)	0.19	0.42 *	1.2% ^↓^	2.2% ^↓^
EccDur (s)	0.52 *	2.42 *	5.4% ^↑^	11.9% ^↑^
ConDur (s)	0.98 **	0.77 *	8.6% ^↑^	7.3% ^↑^
TotDur (s)	0.78 **	1.40 **	6.4% ^↑^	10.4% ^↑^

*,** Significant compared with 24 h at pre-test, *p* < 0.05, *p* < 0.001, increase, ^↑^; decrease, ^↓^; flight time, FT; contact time, CT; flight time–contact time ratio, FT:CT; jump height, JH; peak force, PF; peak power, PP; peak velocity, PV; eccentric duration, EccDur; concentric duration, ConDur; total duration, TotDur, unload countermovement jump, CMJ_unloaded_; load countermovement jump, CMJ_loaded_.

**Table 3:** Correlation with the mRPE score and 0 h, 12 h, and 24 h Countermovement Jump Parameters.

Variable	mRPE		mRPEleg	
	CMJ_Unloaded_	CMJ_Loaded_	CMJ_Unloaded_	CMJ_Loaded_
JH post 0 h	−0.144	−0.383	−0.056	−0.210
JH post 12 h	−0.167	−0.300	−0.129	−0.172
JH post 24 h	−0.220	−0.363	−0.185	−0.248
PP post 0 h	−0.312	−0.411	−0.295	−0.246
PP post 12 h	−0.200	−0.314	−0.371	−0.368
PP post 24 h	−0.219	−0.288	−0.297	−0.322
PV post 0 h	−0.233	−0.543 *	−0.127	−0.225
PV post 12 h	−0.272	−0.425	−0.184	−0.391
PV post 24 h	−0.298	−0.479 *	−0.148	−0.382

* Significant at *p* < 0.05; modified rating of perceived exertion, mRPE; modified rating of perceived exertion leg, mRPEleg; jump height, JH; peak force, PF; peak power, PP; peak velocity, PV; 0 h after countermovement jump test, post 0 h; 12 h after countermovement jump test, post 12 h; 24 h after countermovement jump test, post 24 h.

## Data Availability

The data presented in this study are available on request from the corresponding author.
